# Resolutions on the Death of Dr. H. J. McKellops

**Published:** 1901-06-15

**Authors:** E. G. Betty, J. Taft, Wm. Taft, D. W. Clancy, H. A. Smith, H. T. Smith, C. M. Wright, R. J. Porre

**Affiliations:** Cincinnati; Cincinnati; Cincinnati; Cincinnati; Cincinnati; Cincinnati; Cincinnati; Cincinnati


### RESOLUTIONS ON THE DEATH OF DR. H. J. McKELLOPS.

Whereas, In the providence of the great Creator of the universe He has been pleased to remove from earth's trials and sorrows our beloved friend and co-laborer, Dr. H. J. McKellops, who for many long years has been a devoted worker in the interest of his profession and for the benefit of mankind. He was a constant attendant at the different associations of the profession, especially at the meeting of the Mississippi Valley Association, where his generous features beamed with delight and enthusiasm as he met his friends of years agone. He always brought with him to these meetings something of interest, either in mode of procedure or some valuable appliance, and always took part in the deliberation of its members. He was free in his manner, having no secrets to himself and liberally assisting those who came to him for aid. In his demise the profession has been deprived of a devoted laborer in the vineyard and one who never shirked a duty assigned him. Therefore, be it

*Resolved*, That we cherish in memory the many virtues of our friend, feeling that our loss is his gain, and we may never meet his like on earth again. May his example cheer and inspire us along the pathway of life till the dark future lets its curtain down and we are swallowed up by eternity.

*Resolved*, That although he is forever gone from us we may benefit by what he has done in his love for the profession and his sincere attachment to his friends.

*Resolved*, That we commend his spirit to Him who gave it, and that we may in the future commune with it, freed from the dross of life and purified by the troubles through which it has passed.

*Resolved*, That the secretary be instructed to extend our sincerest sympathy and condolence to the family in this their great bereavement, and that a copy of these resolutions be sent to them and to the editors of the different dental journals of the country.

E. G. Betty, H. A. Smith,
J. Taft, H. T. Smith,
Wm. Taft, C. M. Wright,
D. W. Clancy, R. J. Porre.

Cincinnati, May 30, 1901.

